# Supplementary figures and images for: Impact of the Timing of Maternal Peripartum Depression on Infant Social and Emotional Development at 18 Months
Source: J Clin Med. 2022 Nov 23;11(23):6919. doi: 10.3390/jcm11236919 (PMC9735611; doi:10.3390/jcm11236919)

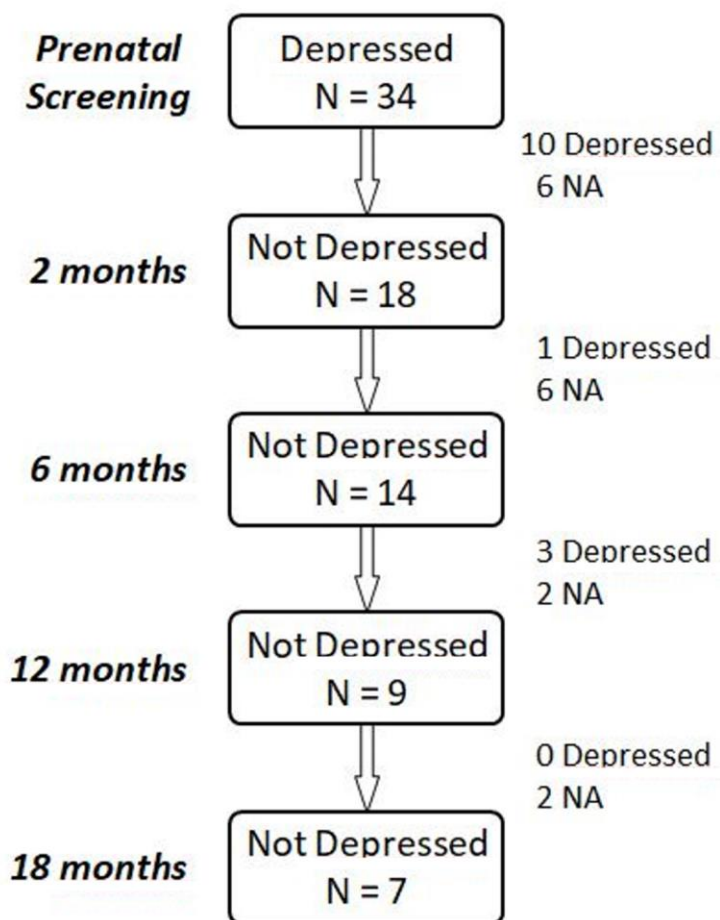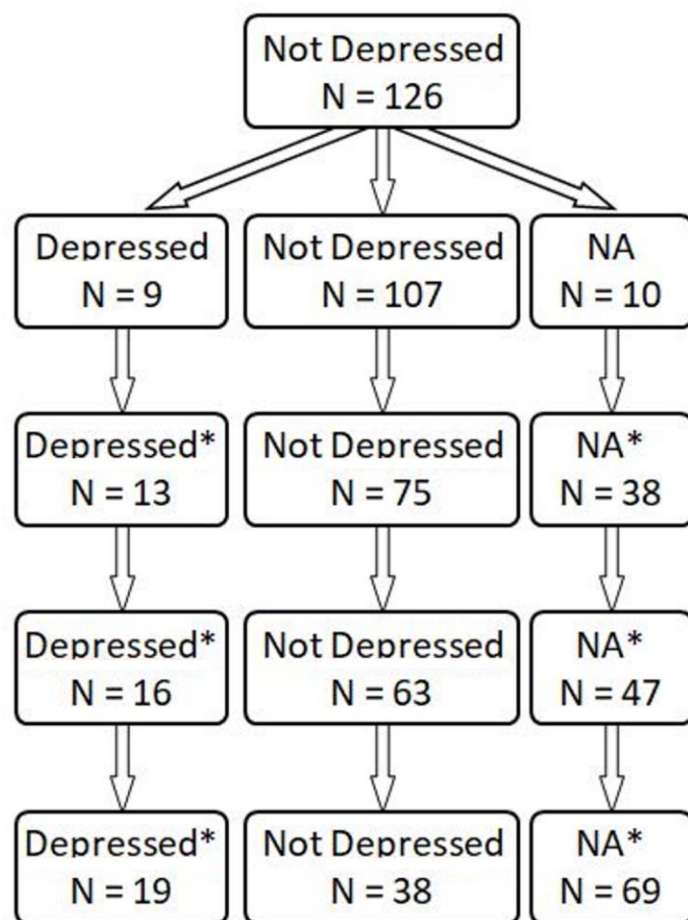

Supplement: Supplementary file 1 [file jcm-11-06919-s001.zip › jcm-2035146-supplementary.pdf]
